# Deep Neural Network Integrated into Network-Based Stratification (D3NS): A Method to Uncover Cancer Subtypes from Somatic Mutations

**DOI:** 10.3390/cancers16162845

**Published:** 2024-08-14

**Authors:** Matteo Valerio, Alessandro Inno, Alberto Zambelli, Laura Cortesi, Domenica Lorusso, Valeria Viassolo, Matteo Verzè, Fabrizio Nicolis, Stefania Gori

**Affiliations:** 1 Medical Oncology, IRCCS Sacro Cuore Don Calabria Hospital, 37024 Negrar di Valpolicella, Verona, Italy; 2 Medical Oncology Unit, IRCCS Istituto Clinico Humanitas and Department of Biomedical Sciences, Humanitas University, 20089 Rozzano, Milan, Italy; alberto.zambelli@hunimed.eu; 3 Oncology, Hematology, and Respiratory Diseases, Azienda Ospedaliera-Universitaria, Policlinico di Modena, 41124 Modena, Italy; 4 Gynecologic Oncology Unit, Humanitas San Pio X, Milan and Humanitas University, Pieve Emanuele, 20090 Milan, Italy; 5 Medical Genetics, Medical Direction, IRCCS Sacro Cuore Don Calabria Hospital, 37024 Negrar di Valpolicella, Verona, Italy; valeria.viassolo@sacrocuore.it; 6 Medical Direction, IRCCS Sacro Cuore Don Calabria Hospital, 37024 Negrar di Valpolicella, Verona, Italy; matteo.verze@sacrocuore.it (M.V.);

**Keywords:** machine learning, deep neural network, autoencoder, somatic mutations, cancer subtypes

## Abstract

**Simple Summary:**

Cancer develops through a complex process involving genetic changes that can lead to uncontrolled cell growth and tumor formation. This research focuses on developing an advanced approach to classify tumors into meaningful subgroups based on somatic mutations. Using machine learning techniques, specifically a deep neural network, and integrating genetic data with known gene interaction networks, we propose a framework for tumor stratification, called D3NS (deep neural network integrated into network-based stratification). This framework identifies patient subtypes predictive for survival and significantly associated with several clinical outcomes (tumor stage, grade and treatment response). We applied D3NS to real-world data from the Cancer Genome Atlas for bladder, ovarian, and kidney cancers. The results demonstrate the potential of this approach to improve cancer stratification, positioning it as a useful base model for cancer research and a promising tool in clinical settings.

**Abstract:**

(1) Background: The identification of tumor subtypes is fundamental in precision medicine for accurate diagnoses and personalized therapies. Cancer development is often driven by the accumulation of somatic mutations that can cause alterations in tissue functions and morphologies. In this work, a method based on a deep neural network integrated into a network-based stratification framework (D3NS) is proposed to stratify tumors according to somatic mutations. (2) Methods: This approach leverages the power of deep neural networks to detect hidden information in the data by combining the knowledge contained in a network of gene interactions, as typical of network-based stratification methods. D3NS was applied using real-world data from The Cancer Genome Atlas for bladder, ovarian, and kidney cancers. (3) Results: This technique allows for the identification of tumor subtypes characterized by different survival rates and significant associations with several clinical outcomes (tumor stage, grade or response to therapy). (4) Conclusion: D3NS can provide a base model in cancer research and could be considered as a useful tool for tumor stratification, offering potential support in clinical settings.

## 1. Introduction

Cancer is a complex disease that arises from a multistep process involving genetic and epigenetic changes, the downregulation of gene expressions, and chromosomal instability. The accumulation of somatic mutations increases with age and can cause alterations in cell growth and functions and in tissue morphologies, promoting cancer development and progression [1,2,3].

Tumors are heterogeneous diseases with variable clinical outcomes. At the molecular level, patients with similar histological and clinical characteristics often show very different genomic aberrations [4,5].

One of the main challenges in cancer informatics is the stratification of tumors into clinically and biologically significant subgroups based on the similarity of molecular profiles. This analysis involves high computational and statistical complexities, requiring increasingly sophisticated algorithms to handle a high number of variables.

Somatic mutation data have unique characteristics, such as high dimensionality and sparsity (prevalence of zero values), distinguishing them from other types of genomic data, such as gene expressions, where for almost every gene, a continuous value is assigned.

In this context, various algorithms based on modern machine learning (ML) techniques have been developed.

Some of these algorithms can be grouped under the category of network-based stratification [6], which integrates somatic mutation profiles with the knowledge of a gene interaction network to classify patients into subtypes. Briefly, the information from each mutation is diffused in the surrounding space defined by the network, propagating the signal to other functionally related genes. This propagation process helps to reduce the sparsity of the data, making them more suitable for stratification. Tumor subtypes are then identified by applying unsupervised ML algorithms, such as NMF (non-negative matrix factorization) and its variants, K-means and DBSCAN (density-based spatial clustering of applications with noise) [6,7,8,9,10].

Other approaches directly use sparse data with unsupervised algorithms derived from artificial neural networks (ANNs), such as autoencoders [11,12].

In these applications, the purpose of the autoencoder is to learn compressed representations of the input data, with which subgroups of patients can then be identified using unsupervised techniques.

With these considerations, in this paper, we propose D3NS, an algorithm for the stratification of tumors based on somatic mutations, which combines the advantages of different techniques: the knowledge contained in a molecular network, the use of an autoencoder to reduce dimensionality and the technique of consensus clustering with K-means to perform a robust stratification of patients into subgroups.

D3NS was applied to three somatic mutation datasets cataloged in The Cancer Genome Atlas (TCGA) for bladder, ovarian and kidney cancers. The algorithm was evaluated for its ability to identify subtypes of cancer significantly associated with survival and major clinical outcomes, such as tumor stage, grade or response to therapy.

## 2. Materials and Methods

### 2.1. Overview of D3NS

D3NS receives as input the set of somatic mutations of a cohort of patients and a network that describes the interactions between genes.

For each patient, somatic mutation data are represented as a binary vector, where each gene is assigned a value of “1” to indicate the presence of a mutation (regardless of the number of mutations) or “0” to indicate its absence.

**Figure 1:**
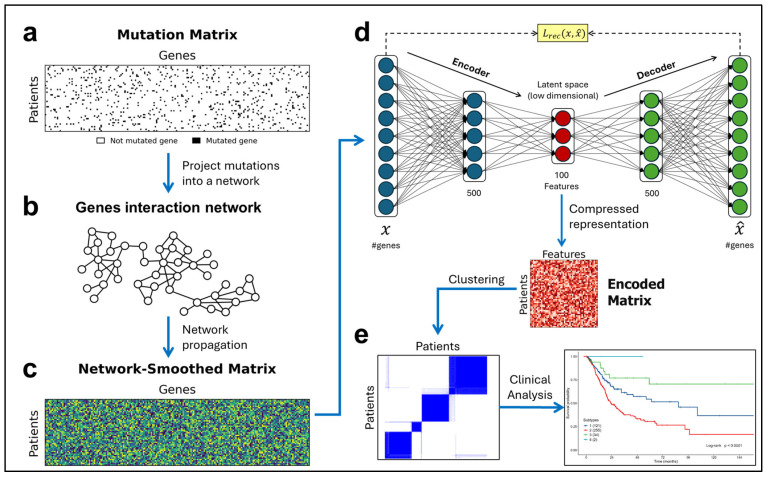
Overview of D3NS. (**a**) Representation of a binary mutation matrix, where for each gene–patient pair, a black point represents a mutated gene corresponding to 1 value. (**b**) Gene interaction network onto which the mutations are projected. (**c**) Representation of network-smoothed matrix with continuous values after the network propagation process. (**d**) Autoencoder’s structure, which receives as input the smoothed mutation profiles of patients and generates their compressed representation, an encoded matrix, with 100 new essential features. (**e**) Subtypes obtained with K-means consensus clustering after 1000 repetitions and the next evaluation based on clinical data.

The set of all the patients (organized in rows) and the set of all the possible mutated genes (arranged in columns) define a binary mutation matrix (MM), Figure 1a, with dimensions patients×genes (genes≫patients), from which, with the integration of a gene network, the algorithm generates a new representation of the mutations useful for achieving better stratification.

This process is divided into the following three phases:

1. Network smoothing: consists of projecting (mapping), for each patient, the binary mutation profile contained in the MM onto a gene interaction network, Figure 1b. Subsequently, the network propagation process [13] is applied to spread the influence of each mutation to the surrounding space related to it. The resulting matrix, the network-smoothed matrix (NSM), will have continuous values and a much lower sparsity compared to that of the initial MM, Figure 1c;
2. Dimensionality reduction: the NSM is provided as input to the autoencoder, which generates its compressed representation, Figure 1d. The result is a matrix with continuous values, an encoded matrix (EM), with dimensions patients×features (features≪genes). The number of features is a parameter of the autoencoder, which defines the number of essential features extracted from the mutations, i.e., the dimension of the latent space where mutations are mapped;
3. Stratification: the consensus clustering technique [14] with the K-means algorithm is applied to the EM, Figure 1e, to stratify patients into subtypes with a variable number of clusters (k = 2 ÷ 6), evaluating their associations with clinical outcomes.

### 2.2. Somatic Mutations and Clinical Data

Somatic mutation information and the related clinical data were retrieved from the public repository cBioPortal for cancer genomics [15] using the pyBioPortal library, specifically created in Python 3 to simplify and automate acquisition, integration, and processing operations.

Three patient cohorts were considered from studies conducted based on specific histological cancer subtypes: muscle-invasive bladder cancer (BLCA) [16], high-grade serous ovarian adenocarcinoma (OVCA) [4], and kidney renal clear cell carcinoma (KIRC) [17]. These studies are cataloged within the TCGA, a well-known research project started in 2006 by the National Cancer Institute and the National Human Genome Research Institute, which has created a database containing a wide variety of cancer data from more than 20,000 samples across 33 types of cancer.

For each dataset, patients with recorded somatic mutations were included. A summary of the distribution of variants is shown in Supplementary Figure S1, and nonsense mutations were excluded.

Table 1 shows the compositions of the three patient cohorts, with the number of genes and the sparsity characterizing the MMs.

Regarding the clinical data, survival times were downloaded in addition to available variables for each study, such as sex, age at diagnosis, tumor stage and grade.

### 2.3. Gene Interaction Networks

The mutations contained in the MM were projected onto a network of gene interactions, the information of which is stored in a database. To assess the effect of the network on the algorithm, three public databases were used: STRING v12.0 [18], HumanNet v3 [19], and Mentha [20].

All the databases considered provide a “confidence score” to define the degree of interaction between the pairs of genes; in this work, only the 10% most confident interactions were retained [6]. After filtering, all the networks were used in an unweighted and undirected mode.

Table 2 summarizes the characteristics of the networks considered in this analysis (filtered values are shown in parentheses).

### 2.4. Network Propagation

After mapping the mutation profile of each patient onto the interaction network, the propagation process, which spreads the mutation signal through the network, is applied.

The propagation process taken as a reference is the one presented in the HotNet2 algorithm [1], called Random Walk with Restart, described by the following Equation (1):

(1) $$F_{t+1}=\alpha F_{t}W+\left(1-\alpha \right)F_{0}$$

where *F*_0_ is the initial binary MM, *F_t_* is the patients×genes mutation matrix at iteration *t*, *W* is a normalized version of the adjacency matrix [13] of the considered network, and α is a tuning parameter with a value between 0 and 1, which controls the length of the diffusion paths along the network and was set at 0.7 [6].

This equation is solved iteratively for different values of *t* until the convergence, defined by the norm $\left\|F_{t+1}-F_{t}\right\|<E$, is reached. At the end of this process, the obtained *F_t_* represents the NSM.

In this work, a simplified version of the previously described propagation process was considered, which still uses the same equation but is solved only once as follows (2):

(2) $$F=\alpha F_{0}W+\left(1-\alpha \right)F_{0}$$

This solution was tested by evaluating the performance compared to that of the iterative version [9] without observing significant differences both in terms of patient stratification and in the association of the generated subtypes with clinical characteristics.

### 2.5. Autoencoder for Dimensionality Reduction

The NSM obtained from network propagation is provided as input to the autoencoder, an unsupervised ML algorithm belonging to the category of ANNs. It consists of two fundamental parts: an encoder and a decoder, as shown in Figure 1d.

During the training phase, the encoder compresses the input data to a “latent space”, obtaining a set of essential features. Subsequently, the decoder attempts to reconstruct the original data from these features.

The primary objective for training the autoencoder is, therefore, not only the accurate reconstruction of the input data but also, above all, the learning of a compact and meaningful representation of the data.

This compression process enables effective dimensionality reduction, allowing for the essential information contained in the data to be represented in a lower-dimensional space.

What distinguishes autoencoders from other dimensionality reduction techniques, such as principal component analysis [21], is their ability to capture complex and nonlinear relationships in the data. Autoencoders are able to leverage nonlinear activation functions and deep neural structures to learn richer and more detailed data representations.

In the simplest case, an autoencoder may have a structure composed of a single hidden layer (between the input and output layers), which number of neurons represents the dimension of the latent space, corresponding to the number of essential features. However, to address more complex problems, it is possible to use architectures typical of deep learning frameworks with multiple hidden layers, Figure 1d.

Each layer of an ANN is characterized by an activation function that determines the behavior of the neurons composing it, giving nonlinear characteristics, to the neural network.

In a given layer (excluding the input layer), each neuron produces an output signal that is dependent on the weighted sum of the signals from the neurons in the previous layer, combined with an activation function that determines the nonlinear behavior, influencing the neural network’s learning process.

In this work, ReLU, rectified linear unit, functions were used for all the hidden layers and the sigmoid function for the output layer, defined respectively by Equations (3) and (4) as follows:

(3) $$f\left(z\right)=max\left(0,z\right)$$

(4) $$f\left(z\right)=\frac{1}{1+e^{-z}}$$

The training process of the autoencoder is based on the objective for minimizing the reconstruction error between the input data and the generated output data, using a loss equation (*L_rec_*) that measures their difference. The loss equation is defined by the mean squared error, as shown in Equation (5):

(5) $$L_{rec}\left(x,\hat{x}\right)=\frac{1}{n}\sum_{i=1}^{n}{\left(x_{i}-{\hat{x}}_{i}\right)}^{2}$$

where *n* is the number of patients, *x* is the input data (NSM), and $\hat{x}$ is the output data (NSM reconstructed).

By minimizing *L_rec_* during training, the autoencoder tries to generate an output that best approximates the original input, capturing the essential features in the data.

The minimization of *L_rec_*, as in all ANNs, involves the use of optimization techniques, such as backpropagation and gradient descent [22], to iteratively calculate and update the network parameters until reaching an acceptable value of *L_rec_* or a certain number of epochs (where an epoch refers to a single iteration of the training process through the entire training set).

Given the complexity and high dimensionality of somatic mutation data, in this paper, an autoencoder with a deep learning architecture was implemented. Its structure comprises three hidden layers composed of 500, 100, and 500 neurons, Figure 1d.

The 100 neurons of the intermediate layer correspond to the dimension of the latent space in which the essential features, which constitute the compact representation of the input data, are defined.

At the end of the training, the mutational profile of each patient is described by these 100 new features, which will compose the EM (patients×features), to be used in the clustering phase.

The implementation of the autoencoder was realized in Python (3.10.12), using the Keras module within the Tensorflow library (2.8.2).

For the training of the autoencoder, the following configuration was set:

1. The input data were split into a training set and a validation set, with a ratio of 90/10, in order to evaluate the algorithm’s performance and prevent overfitting;
2. The Adam algorithm [23] was used to optimize the minimization process of *L_rec_*, setting a learning rate of 0.0001. The learning rate represents the size of the parameter update step of the autoencoder in the procedure for seeking the minimum *L_rec_*;
3. A batch size of 32 was set, useful for accelerating training; it defines the size of the number of samples (patients) processed by the algorithm before updating the parameters;
4. Training was conducted for a maximum of 150 epochs.

### 2.6. K-Means Consensus Clustering

After appropriately scaling the features contained in the EM, the consensus clustering technique [14] combined with the K-means algorithm [24] is applied for the clustering phase to identify the subtypes.

This technique, well-known in cancer research [25], is based on the repetition of sampling and clustering, allowing for the assessment of subtypes’ stabilities with respect to the sampling variability, increasing confidence in their real validity.

The algorithm starts by subsampling a proportion of patients and features from the EM. Each subsampling is then divided into a maximum of k groups using the K-means algorithm. After repeating this process for a specific number of iterations, pairwise consensus values are computed. These values, which indicate the proportion of times two patients were grouped together, are stored in a consensus matrix (CM) for each k value considered.

Finally, to assign each patient to a specific subtype/cluster, hierarchical agglomerative consensus clustering is applied using the distance between the consensus values.

The CM is a square matrix (patients×patients) having patients in both rows and columns, which consensus values range from 0 (for patients never grouped in the same subtype) to 1 (for patients always grouped in the same subtype).

To assess the quality of the identified clusters, it is possible to use heatmaps, graphic representations of the values contained in the CMs, in which a continuous color scale is associated with the range 0–1, such that the value 0 corresponds to white and the value 1 to blue, Figure 1e.

By arranging the values in the CMs so that patients belonging to the same subtype are adjacent, it is possible to obtain, in the ideal case of perfect consensus, heatmaps composed of blue blocks arranged along the diagonal (the identified subtypes) on a white background.

In this work, K-means consensus clustering was implemented using the R package (4.3.2) Consensus Cluster Plus (1.66.0) [26], setting a maximum value for the k clusters to be evaluated equal to 6. The number of repetitions of the K-means algorithm was set at 1000, and for each run, 80% of the patients and 80% of the features were sampled.

### 2.7. Statistical Analysis for Clinical Data

The Kaplan–Meier method and the log-rank test were used to assess the differences between the overall survival (OS) probabilities among the identified patient subtypes.

In both the univariate and multivariate analyses, a Cox proportional hazard model was used to estimate hazard ratios (HRs) and 95% confidence intervals for the subtypes in relation to the OS after verifying the assumption of the proportional hazards.

In particular, for the multivariate analysis, the Cox model was initially built by including all the clinical variables that, in the univariate analysis, reached significance, with at least a *p*-value of <0.2. With the backward stepwise selection technique, the final model was obtained by removing the non-significant variables (*p*-value > 0.05). To evaluate the predictive power added by the identified subtypes, the baseline model, which includes only clinical covariates, was compared with the full model, which includes subtypes in addition to covariates. The likelihood-ratio test was used to compare the two models.

The associations between the subtypes and the available clinical variables were assessed using the Kruskal–Wallis rank sum test or the Wilcoxon rank sum test for continuous variables and Fisher’s exact test or Pearson’s chi-squared test for categorical variables.

For all the tests, a *p*-value of < 0.05 was considered as being statistically significant. The statistical analyses were performed using R statistical software, version 4.3.2 [27].

## 3. Results

The algorithm was tested on three cohorts of patients with bladder, ovarian or kidney cancers, considering three gene interaction networks: STRING v12.0, HumanNet v3, and Mentha.

For each tumor type and each molecular network, EMs were generated and used for patient stratification by applying K-means consensus clustering, considering a number of subtypes/clusters (k) ranging from 2 to 6.

**Figure 2:**
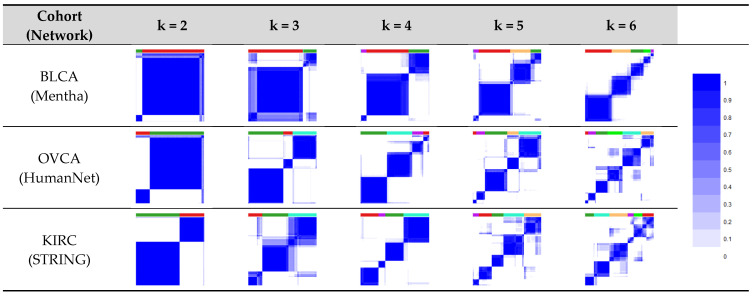
Heatmaps relative to CMs for the different values of k subtypes considered for the stratification, applying some networks to cancer datasets. The blocks in blue correspond to a high consensus value among patient pairs, indicating reliable clusters.

In each of the three cancer types, the proposed algorithm was able to identify structurally robust subtypes, achieving similar results for the three molecular networks, as observed in the heatmaps related to the CMs (Figure 2 and Supplementary Figures S2–S4).

By varying k from 2 to 6, the associations between the identified subtypes and the available clinical variables, particularly survival, were assessed to determine their biological significance.

### 3.1. Bladder Cancer Data

**Figure 3:**
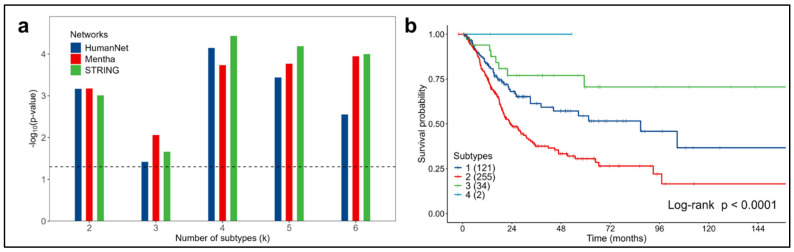
Analysis for bladder cancer in the 412 patients considered from the TCGA dataset. (**a**) Significant associations between survival and subtypes obtained for the three networks considered. Dashed line represents the significance threshold: -log10(Log-rank *p*-value = 0.05). (**b**) OS Kaplan–Meier curves for the four subtypes (k = 4) obtained using the STRING network.

In bladder cancer, each identified subtype was significantly associated with survival (log-rank test *p*-value < 0.05) for all the values of the k subtypes considered and with all the molecular networks (Figure 3a).

The subtypes most significantly associated with survival were obtained using the STRING network for k = 4 (log-rank test *p*-value < 0.0001) (Figure 3b).

Subtype 2 showed the worst prognosis, with a median overall survival of 23.4 months. For subtype 1, a median overall survival time of 86.8 months was observed. Subtypes 3 and 4 displayed the best survival (the median was not reached in the observation interval). The other networks provided similar results (Supplementary Figures S5–S7).

Because subtype 4 consisted of only two patients, setting subtype 3 as a reference in the univariate Cox model, subtype 2 had an HR of 3.60 (95% CI 1.75–7.38, *p*-value < 0.001), while subtype 1 had an HR of 1.99 (95% CI 0.93–4.25, *p*-value = 0.074).

In analyzing the additional clinical characteristics available from the TCGA dataset, significant associations were observed between the four subtypes and the tumor stage. Subtypes 2 and 1 have higher percentages of patients with tumor stage IV (35%) compared to the other subtypes with better survival rates (Table 3).

After evaluating the associations of the clinical variables with survival in the univariate analysis (Supplementary Table S1), a multivariate Cox proportional hazard model was built, including subtypes, age at diagnosis, and tumor stage (Table 4). The predictive power added by the four identified subtypes, when comparing the full model with the baseline model, was significant (likelihood-ratio test *p*-value = 0.0001).

**Figure 4:**
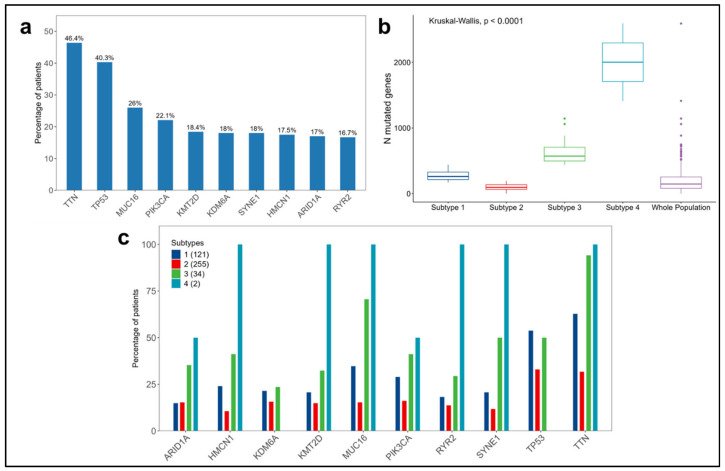
Summary of somatic mutations in the 412 patients considered from the bladder cancer TCGA dataset. (**a**) Distribution of the top 10 mutated genes in the whole population. (**b**) Distributions of the numbers of mutated genes per patient in each subtype and in the whole population. (**c**) Distribution of the top 10 mutated genes across the subtypes.

Figure 4a provides an overview of the top 10 mutated genes in the entire population considered from the bladder cancer TCGA dataset (N = 412). Their distributions across the four subtypes are represented in Figure 4c. In the overall population, the most frequently mutated genes are *TTN* and *TP53*, present in 46.4% and 40.3% of the patients, respectively. A similar pattern is observed in each subtype, except for subtype 4, which did not show mutations in *TP53*; however, this subtype accounts for only two patients.

We observed significant differences (*p*-value < 0.0001) in the numbers of mutated genes per patient among the subtypes (Figure 4b and Supplementary Table S2). Subtypes 1 and 2 are characterized by lower numbers of mutated genes, with medians of 261 and 95, respectively, compared to subtypes 3 and 4, which displayed significantly higher medians of 572 and 2002, respectively.

### 3.2. Ovarian Cancer Data

**Figure 5:**
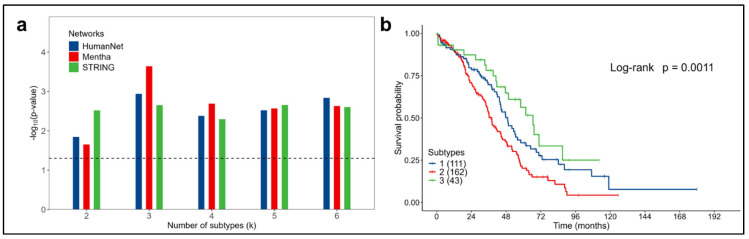
Analysis for ovarian cancer in the 316 patients considered from the TCGA dataset. (**a**) Significant associations between survival and subtypes obtained for the three networks considered. Dashed line represents the significance threshold: -log10(Log-rank *p*-value = 0.05). (**b**) OS Kaplan–Meier curves for the three subtypes (k = 3) obtained using the HumanNet network.

In the initial analysis, the associations between the identified subtypes and survival were assessed, yielding significant results for all the values of the k clusters considered and with all the molecular networks (log-rank test *p*-value < 0.05), Figure 5a.

Considering three subtypes (k = 3) using the HumanNet network, the Kaplan–Meier curves obtained (log-rank test *p*-value = 0.0011), reported in Figure 5b, showed that patients with subtype 2 ovarian cancer had the most aggressive disease (with a median overall survival of 38 months) compared to the less aggressive subtype 3 (with a median overall survival of 66.6 months). An overview of the results obtained with the other networks is available in Supplementary Figures S8–S10.

Setting subtype 3 as the reference in the univariate Cox proportional hazard model, subtype 1 had an HR of 1.42 (95% CI 0.84–2.38, *p*-value = 0.19), while subtype 2 had an HR of 2.17 (95% CI 1.33–3.55, *p*-value = 0.002).

Among the identified subtypes and the other clinical variables available from the TCGA dataset (Table 5), significant associations were observed with the tumor stage, age at diagnosis, and response to platinum therapy after surgery. Patients in subtype 2, who have a lower survival rate, are all in stage III-IV (except for one case) compared to the other subtypes, which have higher percentages of patients in stage II. Regarding the responses to platinum therapy, patients in subtype 3 had the highest percentage of complete responses (86%) without any cases of progression or stable disease, which corresponds to the best survival rate compared to those of the other subtypes.

The associations between the clinical variables and survival are detailed in Supplementary Table S3.

In the multivariate analysis, the subtypes, age at diagnosis, and response to platinum therapy were significantly associated with survival, as shown in the final Cox model (Table 6). The predictive power added by the subtypes, compared to the baseline model, was significant (likelihood-ratio test *p*-value = 0.01).

**Figure 6:**
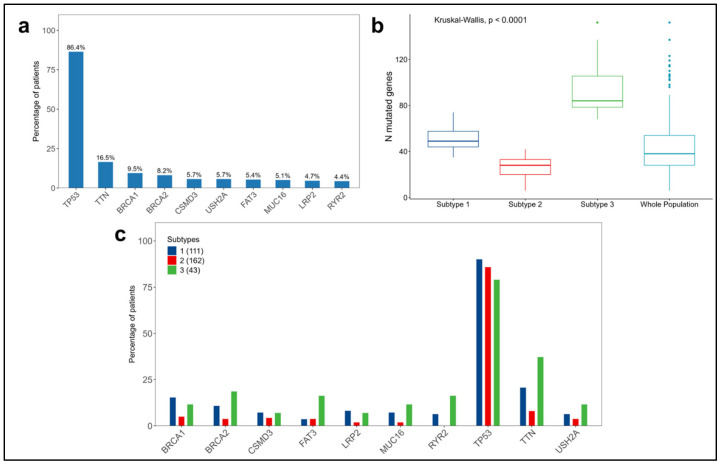
Summary of somatic mutations in the 316 patients considered from the ovarian cancer TCGA dataset. (**a**) Distribution of the top 10 mutated genes in the whole population. (**b**) Distributions of the numbers of mutated genes per patient in each subtype and in the whole population. (**c**) Distribution of the top 10 mutated genes across the subtypes.

Regarding somatic mutations, in the entire population considered from the ovarian cancer TCGA dataset (N = 316), *TP53* dominates over all the other genes, with a prevalence of 86.4%, followed by *TTN*, *BRCA1*, and *BRCA2* to a much lower extent (Figure 6a). *BRCA1* and *BRCA2* have been investigated in various studies highlighting their significant prognostic and predictive roles in both survival and sensitivity to platinum-based treatments. *BRCA1* and *BRCA2* have higher prevalences in subtypes 1 and 3 than in subtype 2 (Figure 6c).

Finally, regarding the distribution of the number of mutated genes per patient, significant differences were observed between the subtypes (*p*-value < 0.0001), with subtype 2 having the lowest number of mutated genes, characterized by a median of 28 mutations compared to 49 and 84 in subtypes 1 and 3, respectively (Figure 6b and Supplementary Table S4).

### 3.3. Kidney Cancer Data

**Figure 7:**
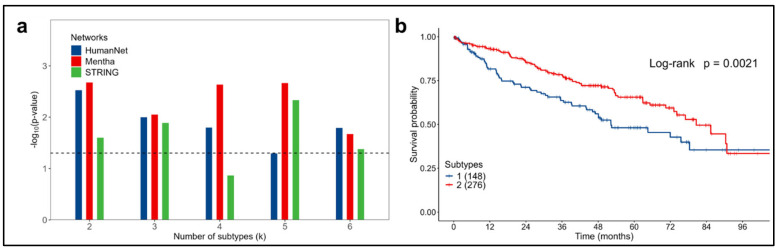
Analysis for kidney cancer in the 424 patients considered from the TCGA dataset. (**a**) Significant associations between survival and subtypes obtained for the three networks considered. Dashed line represents the significance threshold: -log10(Log-rank *p*-value = 0.05). (**b**) OS Kaplan–Meier curves for the two subtypes (k = 2) obtained using the Mentha network.

In kidney cancer, for each value of the k cluster and for almost all the molecular networks considered, subtypes significantly associated with survival were identified (log-rank test *p*-value < 0.05), as shown in Figure 7a.

With the Mentha network for k = 2, the subtypes most significantly associated with survival were identified (log-rank test *p*-value = 0.0021), Figure 7b. Patients were classified into a high-risk group (subtype 1) or a low-risk group (subtype 2), with median overall survivals of 52.2 and 80.6 months, respectively, and an HR of 1.68 (95% CI 1.20–2.34, *p*-value = 0.002) for subtype 1 vs. subtype 2. For all the other results, refer to Supplementary Figures S11–S13.

In analyzing the clinical variables available from the TCGA (Table 7), a significant association was observed between the subtypes and age at diagnosis. Patients in subtype 2 had a median age of 59 years, whereas those in subtype 1 had a median age of 64 years. No significant differences were found between the subtypes for the other variables.

In the univariate analysis, all the covariates were significantly associated with survival, except for sex (Supplementary Table S5).

The identified subtypes were significant predictors of survival, adding predictive power to the baseline Cox model, which includes covariates only, such as the tumor stage, grade and age at diagnosis (likelihood-ratio test *p*-value = 0.02), Table 8.

**Figure 8:**
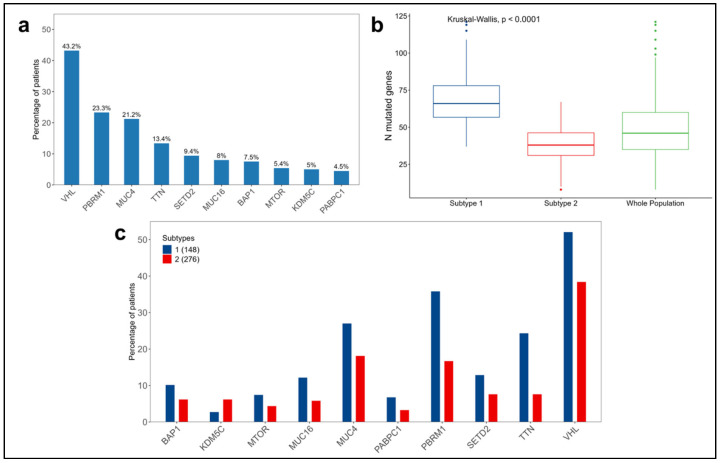
Summary of somatic mutations in the 424 patients considered from the kidney cancer TCGA dataset. (**a**) Distribution of the top 10 mutated genes in the whole population. (**b**) Distributions of the numbers of mutated genes per patient in each subtype and in the whole population. (**c**) Distribution of the top 10 mutated genes across the subtypes.

Regarding somatic mutations, *VHL* has the highest prevalence (43.2%) in the entire population considered from the kidney cancer TCGA dataset (N = 424), followed by *PBRM1* and *MUC4*, both slightly above 20% (Figure 8a). The distributions of the mutations across the subtypes are shown in Figure 8c.

Comparing the subtypes by the number of mutated genes per patient, a significant difference was observed (*p*-value < 0.0001), with subtype 1 having a median of 66 mutated genes compared to 38 in subtype 2 (Figure 8b and Supplementary Table S6).

## 4. Discussion

In the expanding landscape of genomic algorithms for tumor subtype characterization, the approach proposed in this study combines the strengths of various methodologies.

The proposed algorithm leverages prior knowledge from molecular networks, the power of deep neural networks in extracting hidden information from data, and the robustness of consensus clustering in revealing stable clusters within the data.

Most studies applying stratification algorithms mainly utilize continuous genomic data formats, such as gene expressions and other omics profiles. In contrast, D3NS identifies significant subtypes, both biologically and clinically, by analyzing somatic mutations encoded in binary format, categorizing each gene as mutated or not mutated.

The incorporation of molecular networks, with constantly updated databases, not only addresses the challenge of mutation sparsity but also enhances significant biological foundations essential for effective subtype classification.

The autoencoder, implemented with a deep learning architecture, mitigates high data dimensionality inherent in large gene datasets, yielding a compressed representation that retains comprehensive input information. This avoids the need for feature selection algorithms, which often restrict the informative range essential for stratification.

From a stratification perspective, the algorithm delivers well-defined clusters at the structural level, as shown by the heatmaps (Figure 2 and Supplementary Figures S2–S4), which are very similar to those obtained by other state-of-the-art algorithms for tumor subtype discovery based on somatic mutations [6,7,8,9,10].

Our results demonstrate D3NS’s capability in identifying predictive subtypes for survival (Figure 3, Figure 5 and Figure 7) and their associations with other clinical variables, such as tumor stage, grade or treatment response, confirming its validity across diverse molecular networks. Furthermore, integrating subtype information with clinical variables significantly enhances the predictive power for survival, suggesting these subtypes as molecular prognostic indicators.

This study considered datasets that include only the most common histological subtypes for each tumor localization. For bladder cancer, cases with muscle-invasive bladder cancer, both non-papillary and papillary without other histological characterizations, were included (Table 3); for ovarian cancer, the dataset focused on high-grade serous carcinoma [4], and for kidney cancer, the data refer to clear cell renal cell carcinoma [17]. This approach allowed for us to explore the molecular variability within a homogeneous group of cases, identifying subtypes with significantly different characteristics.

Several studies in serous ovarian cancer highlight the prognostic and predictive roles of *BRCA1* and *BRCA2* germlines and somatic mutations in survival and responses to platinum-based treatments [28,29,30,31,32,33,34]. Consistently, our findings on ovarian cancer reveal higher prevalences of *BRCA1* and *BRCA2* mutations in subtypes 1 and 3, which are associated with better survival and responses to treatments compared to subtype 2 (Figure 6c).

Among the top 10 mutated genes found in the kidney cancer dataset, *PBRM1*, *BAP1*, *SETD2*, and *VHL* have been implicated in tumor progression and poor prognoses [35]. As shown in Figure 8c, these mutated genes have a higher prevalence in subtype 1, correlating with worse prognoses.

Although this algorithm was tested on three datasets, its application may be extended to other tumor localizations and, within bladder, ovarian, and kidney cancers, to a wider variety of histological cancer subtypes through parameter adjustments to optimize the performance. These broader applications could potentially be used to explore the concordance between tumor subtypes identified using the algorithm and the more classical histological tumor stratification, providing further insights into the definition of tumor pathological mechanisms.

Although this research proposes a powerful methodology for tumor stratification, it has some limitations.

First, to ascertain the robustness and generalizability of the D3NS, future studies should validate its application to diverse real-world datasets of similar tumor types, including comparisons with other methods already available in the literature. Second, further analyses are needed to investigate the genetic compositions of the subtypes, given the significant difference observed in the numbers of mutated genes among them.

Additionally, this study investigated neither specific mutation classes (e.g., nonsense mutations) nor epigenetic alterations (e.g., DNA methylation and histone modifications), which potentially impact tumor suppressor genes.

Despite these limitations, we believe that the development of more sophisticated versions of the autoencoder and the use of other stratification approaches could help to better capture the biological complexities hidden in genomic data.

## 5. Conclusions

Identifying tumor subtypes is crucial for precise diagnoses and personalized therapies. Our study demonstrates that the D3NS algorithm is a valuable tool for tumor stratification in the context of precision medicine.

Integrating somatic gene panel testing with D3NS analysis can offer potential support in clinical settings, with opportunities for improvement through the selection of clinically relevant genes and appropriate gene interaction networks.

In conclusion, this approach can provide a base model in cancer research, adaptable for different types of cancer through necessary adjustments.

## Figures and Tables

**Table 1:** Summary of the three cohorts of patients.

Cohort	N Patients	N Genes	MM Sparsity
Bladder cancer (BLCA)	412	16,385	98.8%
Ovarian cancer (OVCA)	316	7961	99.5%
Kidney cancer (KIRC)	424	10,257	99.5%

**Table 2:** Summary of gene interaction networks.

Network	N Nodes ^1^	N Edges ^2^	Link
STRING v12.0 [18]	19,622 (12,030) ^3^	6,857,702 (91,983) ^3^	https://string-db.org accessed on 18 March 2024
HumanNet v3 [19]	18,449 (15,435)	977,483 (97,737)	https://www.inetbio.org/humannet/ accessed on 18 March 2024
Mentha [20]	18,861 (8176)	339,047 (26,584)	https://mentha.uniroma2.it/download.php accessed on 18 March 2024

^1^ N genes; ^2^ N interactions; ^3^ filtering was performed on the set of interactions categorized at least as “medium confidence”, meaning, having a score of ≥ 0.4.

**Table 3:** Bladder cancer distributions of clinical characteristics in the four subtypes (k = 4) obtained using the STRING network.

			Subtype (k)				
Characteristic	N	Overall 412 (100%)	1 121 (29%)	2 255 (62%)	3 34 (8.3%)	4 2 (0.5%)	*p*-Value ^1^
Sex	411						0.2
Female		108 (26%)	24 (20%)	74 (29%)	10 (29%)	0 (0%)	
Male		303 (74%)	96 (80%)	181 (71%)	24 (71%)	2 (100%)	
*Missing*		1	1	0	0	0	
Age at Diagnosis	411	69 (60; 76)	69 (61; 76)	68 (60; 76)	69 (61; 76)	73 (71; 74)	>0.9
*Missing*		1	1	0	0	0	
Weight	368	78 (65; 92)	80 (65; 94)	77 (65; 90)	82 (72; 95)	108 (101; 114)	0.2
*Missing*		44	17	23	4	0	
Tumor Stage	409						**0.031**
I–II		133 (33%)	42 (35%)	79 (31%)	11 (32%)	1 (50%)	
III		141 (34%)	36 (30%)	85 (34%)	19 (56%)	1 (50%)	
IV		135 (33%)	42 (35%)	89 (35%)	4 (12%)	0 (0%)	
*Missing*		3	1	2	0	0	
Grade	408						0.2
High Grade		387 (95%)	115 (97%)	236 (93%)	34 (100%)	2 (100%)	
Low Grade		21 (5.1%)	3 (2.5%)	18 (7.1%)	0 (0%)	0 (0%)	
*Missing*		4	3	1	0	0	
Histological Subtype	406						0.6
Non-Papillary		273 (67%)	76 (64%)	174 (69%)	22 (67%)	1 (50%)	
Papillary		133 (33%)	43 (36%)	78 (31%)	11 (33%)	1 (50%)	
*Missing*		6	2	3	1	0	

N is the number of non-missing values; continuous variables are expressed as medians (IQRs) and categorical variables as n (%); ^1^ Kruskal–Wallis rank sum test; Fisher’s exact test; Pearson’s chi-squared test; bold values indicate *p*-values of < 0.05.

**Table 4:** Multivariable Cox proportional hazard model for OS (bladder cancer data with k = 4).

Characteristic (N Observations = 407; N Events = 178)	HR ^1^	95% CI ^1^	*p*-Value	*p*-Value ^2^ Global
Subtype (k)				**<0.001**
1	1.76	0.82, 3.80	0.148	
2	3.10	1.50, 6.42	**0.002**	
3 *(Reference)*	—	—		
4	0	—, —	0.994	
Age at Diagnosis	1.03	1.01, 1.04	**<0.001**	
Tumor Stage				**<0.001**
I–II *(Reference)*	—	—		
III	1.55	1.01, 2.36	**0.045**	
IV	2.62	1.76, 3.89	**<0.001**	

^1^ HR = hazard ratio; CI = confidence interval; ^2^ global *p*-values for categorical variables with more than two categories; bold values indicate *p*-values of < 0.05.

**Table 5:** Ovarian cancer distributions of clinical characteristics in the three subtypes (k = 3) obtained using the HumanNet network.

			Subtype (k)			
Characteristic	N	Overall 316 (100%)	1 111 (35%)	2 162 (51%)	3 43 (14%)	*p*-Value ^1^
Age at Diagnosis	316	59 (51; 69)	57 (50; 66)	59 (51; 70)	63 (59; 72)	**0.043**
Tumor Stage	315					**0.007**
II		14 (4.4%)	9 (8.2%)	1 (0.6%)	4 (9.3%)	
III		248 (79%)	83 (75%)	134 (83%)	31 (72%)	
IV		53 (17%)	18 (16%)	27 (17%)	8 (19%)	
*Missing*		1	1	0	0	
Grade	309					0.7
G2		28 (9.1%)	12 (11%)	12 (7.6%)	4 (9.3%)	
G3		281 (91%)	97 (89%)	145 (92%)	39 (91%)	
*Missing*		7	2	5	0	
Residual Tumor After Surgery	278					0.6
>10 mm		70 (25%)	27 (29%)	33 (23%)	10 (25%)	
≤10 mm		208 (75%)	67 (71%)	111 (77%)	30 (75%)	
*Missing*		38	17	18	3	
Response to Platinum Therapy	260					**0.030**
Complete Response		184 (71%)	63 (71%)	91 (67%)	30 (86%)	
Partial Response		39 (15%)	13 (15%)	21 (15%)	5 (14%)	
Progressive Disease		25 (9.6%)	12 (13%)	13 (9.6%)	0 (0%)	
Stable Disease		12 (4.6%)	1 (1.1%)	11 (8.1%)	0 (0%)	
*Missing*		56	22	26	8	

N is the number of non-missing values; continuous variables are expressed as medians (IQRs) and categorical variables as n (%); ^1^ Kruskal–Wallis rank sum test; Fisher’s exact test; Pearson’s chi-squared test; bold values indicate *p*-values of < 0.05.

**Table 6:** Multivariable Cox proportional hazard model for OS (ovarian cancer data with k = 3).

Characteristic (N Observations = 260; N Events = 144)	HR ^1^	95% CI ^1^	*p*-Value	*p*-Value ^2^ Global
Subtype (k)				**0.011**
1	1.35	0.74, 2.45	0.323	
2	2.03	1.15, 3.58	**0.014**	
3 *(Reference)*	—	—		
Age at Diagnosis	1.02	1.01, 1.04	**0.004**	
Response After Platinum Therapy				**<0.001**
Complete Response *(Reference)*	—	—		
Partial Response	4.20	2.64, 6.68	**<0.001**	
Progressive Disease	5.46	3.31, 9.01	**<0.001**	
Stable Disease	2.78	1.31, 5.91	**0.008**	

^1^ HR = hazard ratio; CI = confidence interval; ^2^ global *p*-values for categorical variables with more than two categories; bold values indicate *p*-values of < 0.05.

**Table 7:** Kidney cancer distributions of clinical characteristics in the two subtypes (k = 2) obtained using the Mentha network.

			Subtype (k)		
Characteristic	N	Overall 424 (100%)	1 148 (35%)	2 276 (65%)	*p*-Value ^1^
Sex	424				0.7
Female		147 (35%)	53 (36%)	94 (34%)	
Male		277 (65%)	95 (64%)	182 (66%)	
Age at Diagnosis	424	61 (52; 70)	64 (57; 73)	59 (50; 69)	**<0.001**
Tumor Stage	423				0.3
I–II		241 (57%)	78 (53%)	163 (59%)	
III		112 (26%)	46 (31%)	66 (24%)	
IV		70 (17%)	24 (16%)	46 (17%)	
*Missing*		1	0	1	
Grade	423				0.4
G1–G2		181 (43%)	58 (39%)	123 (45%)	
G3		175 (41%)	67 (46%)	108 (39%)	
G4		67 (16%)	22 (15%)	45 (16%)	
*Missing*		1	1	0	

N is the number of non-missing values; continuous variables are expressed as medians (IQRs) and categorical variables as n (%); ^1^ Wilcoxon rank sum test; Pearson’s chi-squared test; bold values indicate *p*-values of < 0.05.

**Table 8:** Multivariable Cox proportional hazard model for OS (kidney cancer data with k = 2).

Characteristic (N Observations = 419; N Events = 140)	HR ^1^	95% CI ^1^	*p*-Value	*p*-Value ^2^ Global
Subtype (k)				
1	1.51	1.06, 2.14	**0.022**	
2 *(Reference)*	—	—		
Age at Diagnosis	1.04	1.02, 1.05	**<0.001**	
Tumor Stage				**<0.001**
I–II *(Reference)*	—	—		
III	2.24	1.44, 3.47	**<0.001**	
IV	5.16	3.26, 8.17	**<0.001**	
Grade				**0.013**
G1–G2 *(Reference)*	—	—		
G3	1.17	0.77, 1.79	0.457	
G4	2.02	1.24, 3.29	**0.005**	

^1^ HR = hazard ratio; CI = confidence interval; ^2^ global *p*-values for categorical variables with more than two categories; bold values indicate *p*-values of < 0.05.

## Data Availability

The data used in this research are available online from the cBioPortal website (https://www.cbioportal.org/). Additionally, the data can be accessed programmatically with Python 3 using the pyBioPortal library. More information and documentation can be found at https://github.com/Matteo-Valerio/pyBioPortal.

## Supplementary Materials

The following supporting information can be downloaded at: https://www.mdpi.com/article/10.3390/cancers16162845/s1, Figure S1: Summary of the distribution of variants in the entire population in the (a) bladder cancer dataset, (b) ovarian cancer dataset and (c) kidney cancer dataset; Figure S2: Heatmaps for bladder cancer patients relative to CMs for the different values of k considered for the stratification; Figure S3: Heatmaps for ovarian cancer patients relative to CMs for the different values of k considered for the stratification; Figure S4: Heatmaps for kidney cancer patients relative to CMs for the different values of k considered for the stratification; Figure S5: Kaplan–Meier survival curves for bladder cancer patients for different values of k with STRING network; Figure S6: Kaplan–Meier survival curves for bladder cancer patients for different values of k with HumanNet network; Figure S7: Kaplan–Meier survival curves for bladder cancer patients for different values of k with Mentha network; Figure S8: Kaplan–Meier survival curves for ovarian cancer patients for different values of k with STRING network; Figure S9: Kaplan–Meier survival curves for ovarian cancer patients for different values of k with HumanNet network; Figure S10: Kaplan–Meier survival curves for ovarian cancer patients for different values of k with Mentha network; Figure S11: Kaplan–Meier survival curves for kidney cancer patients for different values of k with STRING network; Figure S12: Kaplan–Meier survival curves for kidney cancer patients for different values of k with HumanNet network; Figure S13: Kaplan–Meier survival curves for kidney cancer patients for different values of k with Mentha network; Table S1: Univariable Cox proportional hazard models of the characteristics for OS (BLCA data with k = 4); Table S2: Distribution of the number of mutated genes per patient in each subtype and in the whole population for bladder cancer, with k = 4; Table S3: Univariable Cox proportional hazard models of the characteristics for OS (OVCA data with k = 3); Table S4: Distribution of the number of mutated genes per patient in each subtype and in the whole population for ovarian cancer, with k = 3; Table S5: Univariable Cox proportional hazard models of the characteristics for OS (KIRC data with k = 2); Table S6: Distribution of the number of mutated genes per patient in each subtype and in the whole population for kidney cancer, with k = 2.
