# Supplementary material for: Deep Neural Network Integrated into Network-Based Stratification (D3NS): A Method to Uncover Cancer Subtypes from Somatic Mutations
Source: Cancers (Basel). 2024 Aug 14;16(16):2845. doi: 10.3390/cancers16162845 (PMC11352240; doi:10.3390/cancers16162845)
Supplement: Supplementary file 1 [file cancers-16-02845-s001.zip › cancers-3139691-supplementary.pdf]

Supplementary Materials

Supplementary Figure S1. Summary of the distribution of variants in the entire population in the (a) bladder cancer dataset, (b) ovarian cancer dataset and (c) kidney cancer dataset.

a

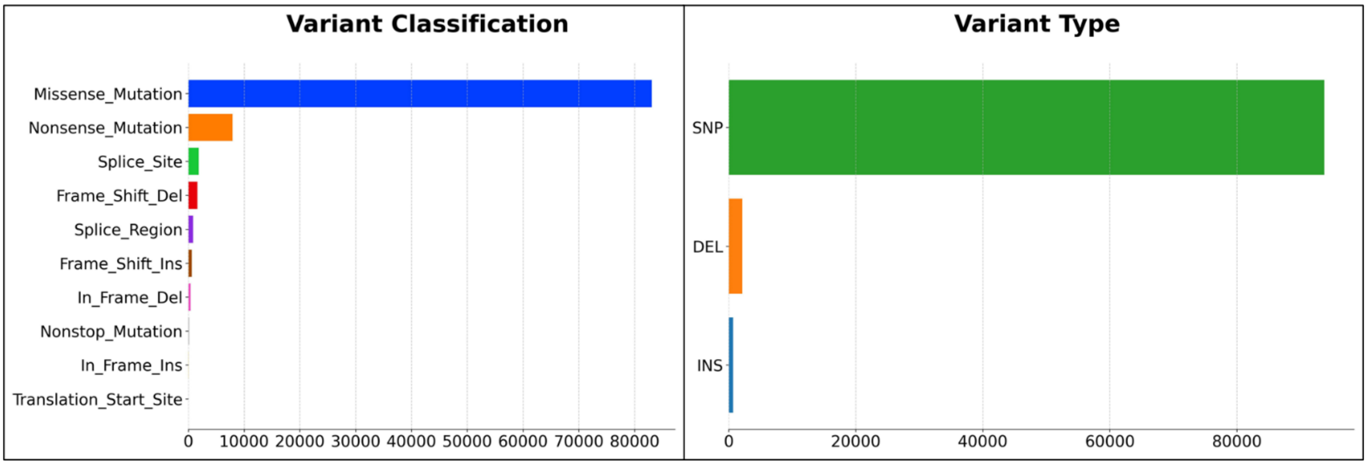

b

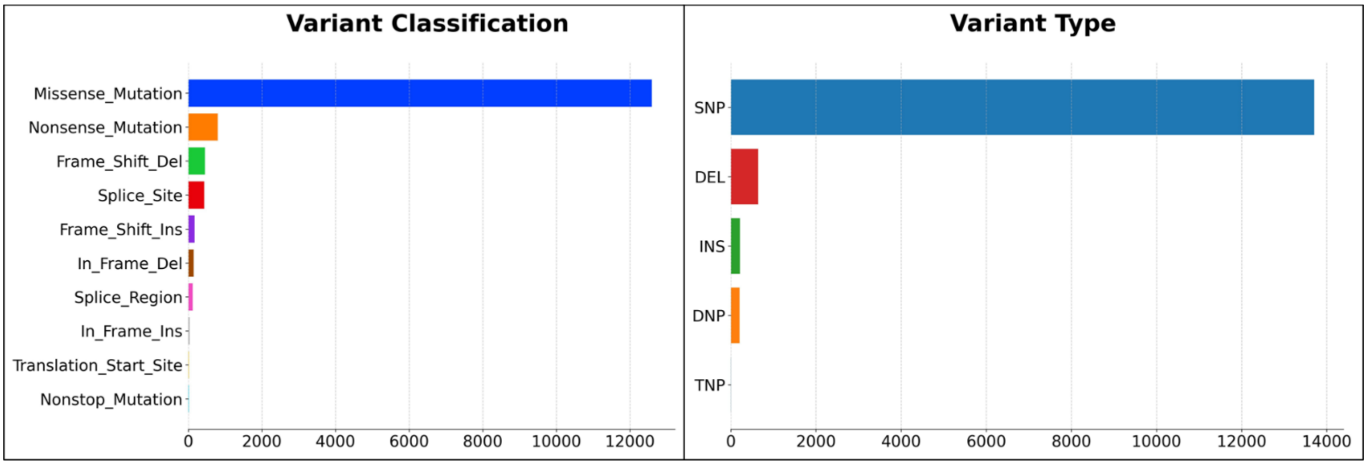

c

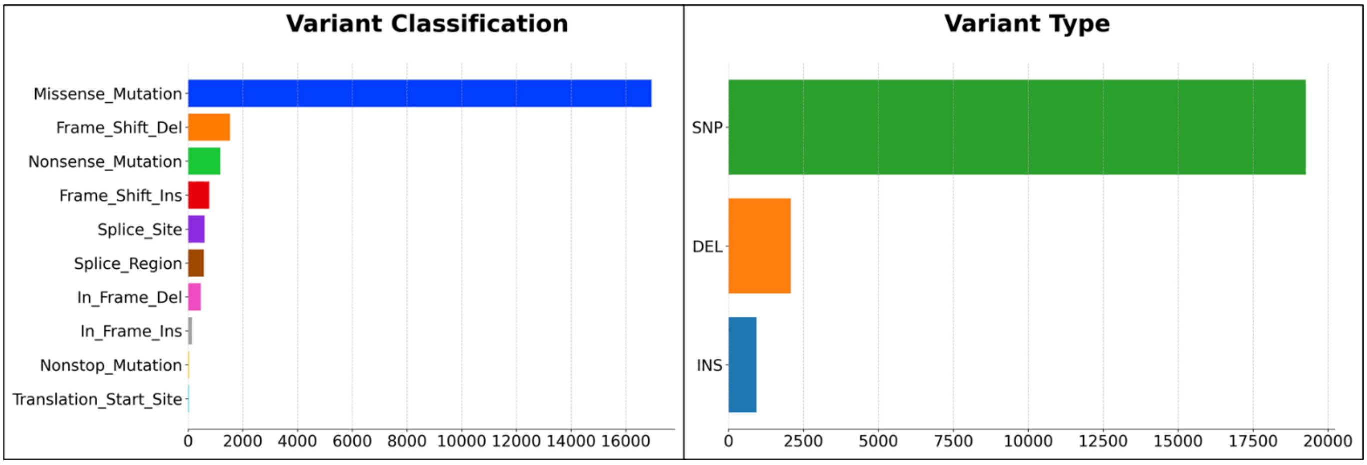

Supplementary Figure S2. Heatmaps for bladder cancer patients relative to CMs for the different values of k considered for the stratification.

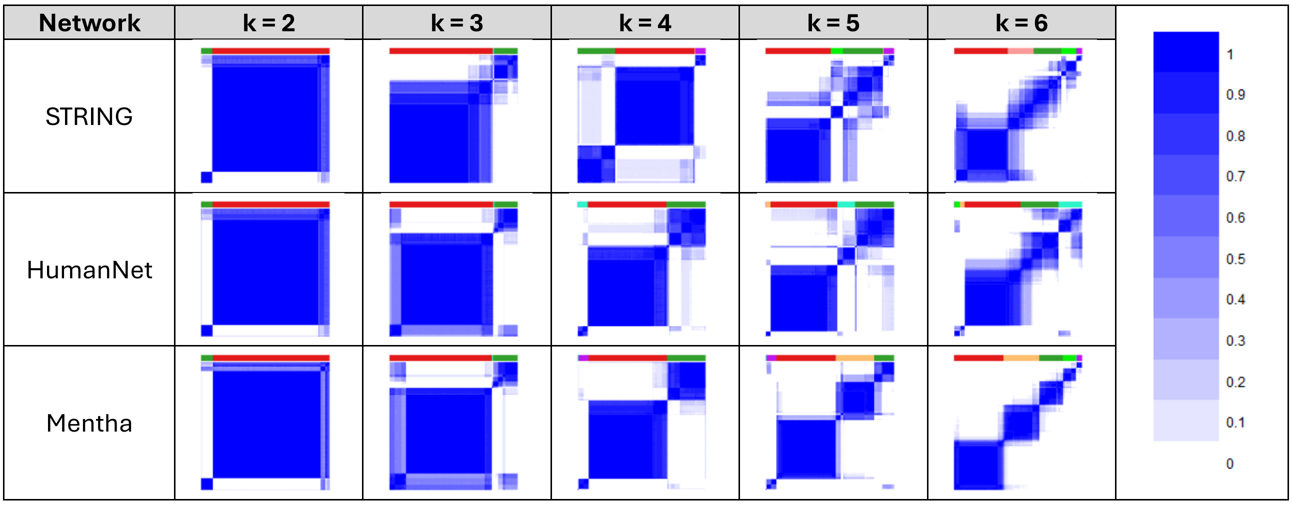

Supplementary Figure S3. Heatmaps for ovarian cancer patients relative to CMs for the different values of k considered for the stratification.

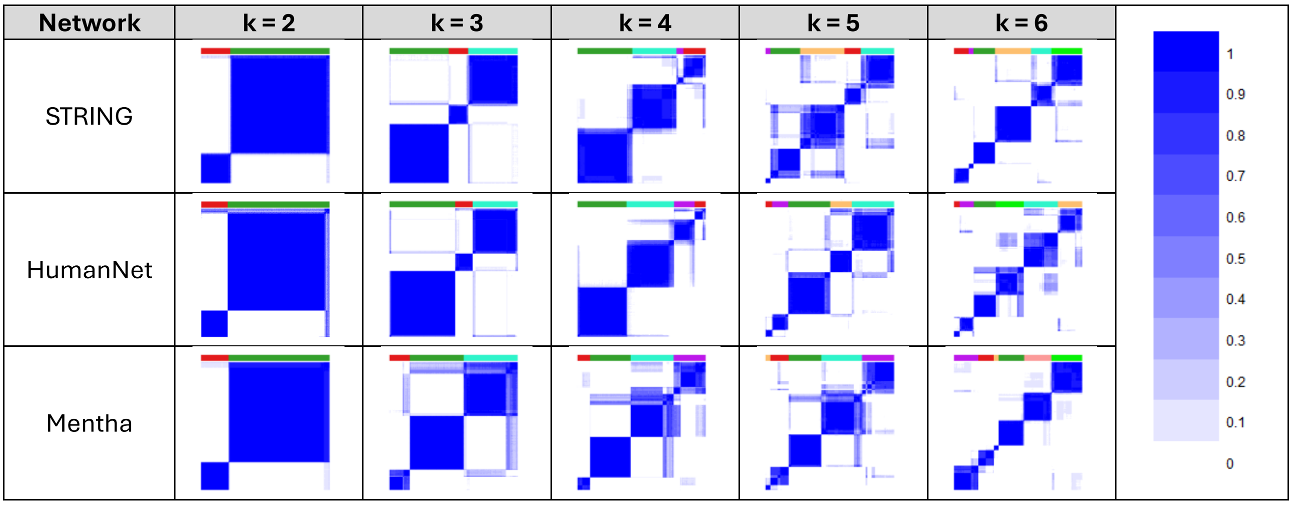

Supplementary Figure S4. Heatmaps for kidney cancer patients relative to CMs for the different values of k considered for the stratification.

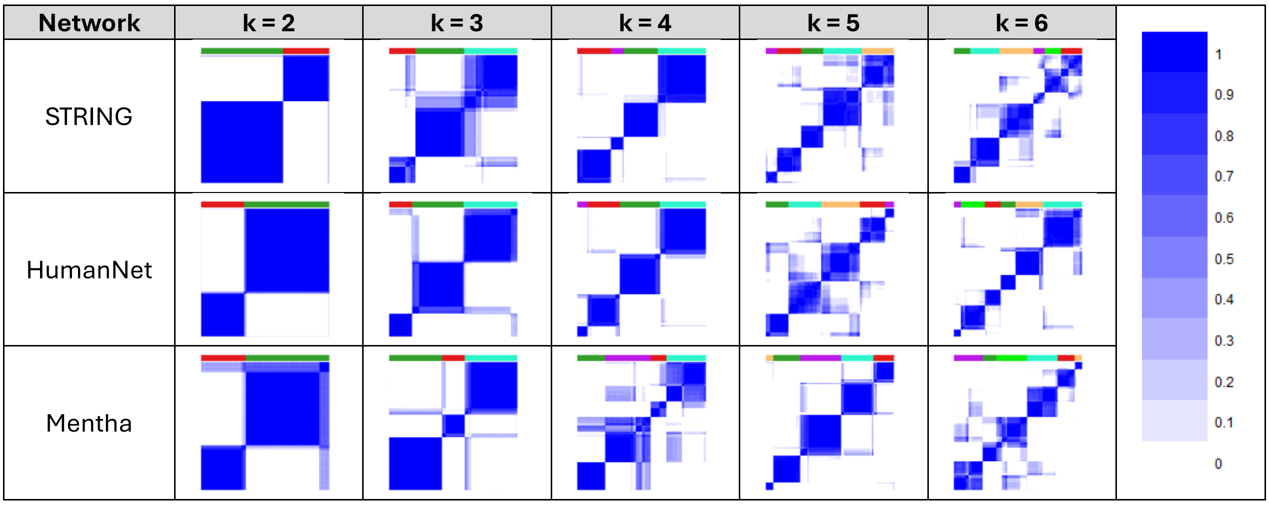

Supplementary Figure S5. Kaplan–Meier survival curves for bladder cancer patients for different values of k with STRING network.

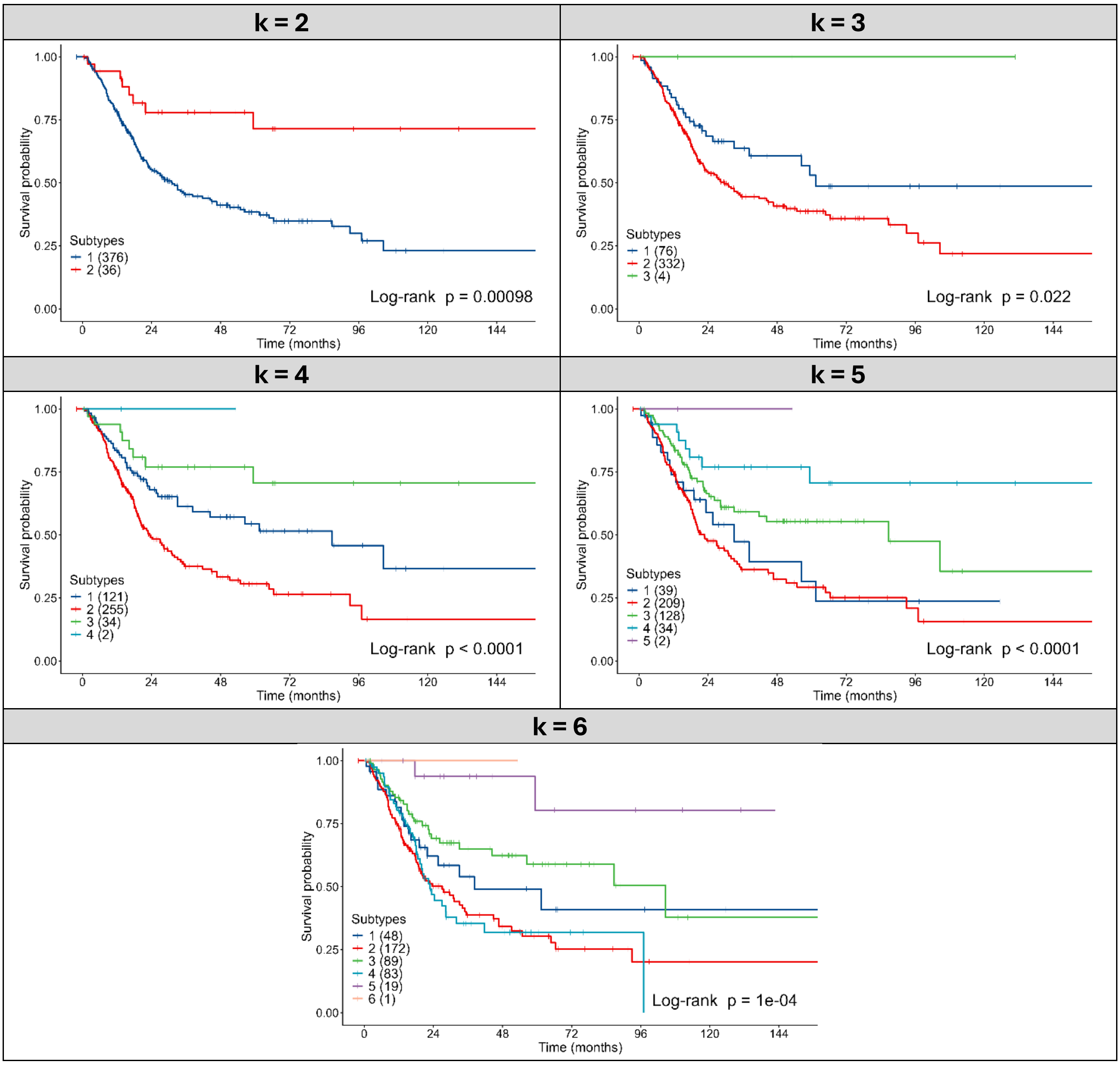

Supplementary Figure S6. Kaplan–Meier survival curves for bladder cancer patients for different values of k with HumanNet network.

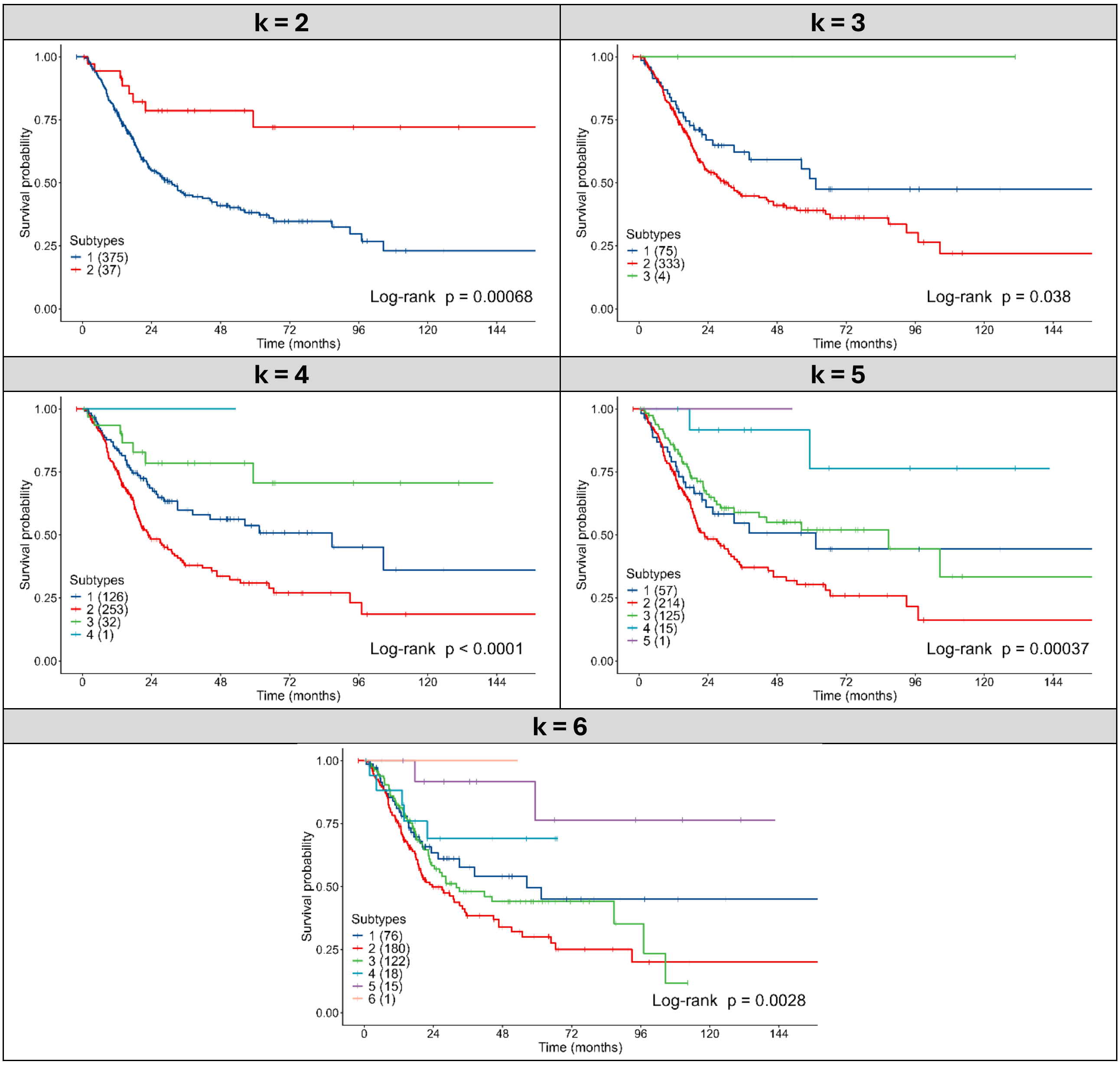

Supplementary Figure S7. Kaplan–Meier survival curves for bladder cancer patients for different values of k with Mentha network.

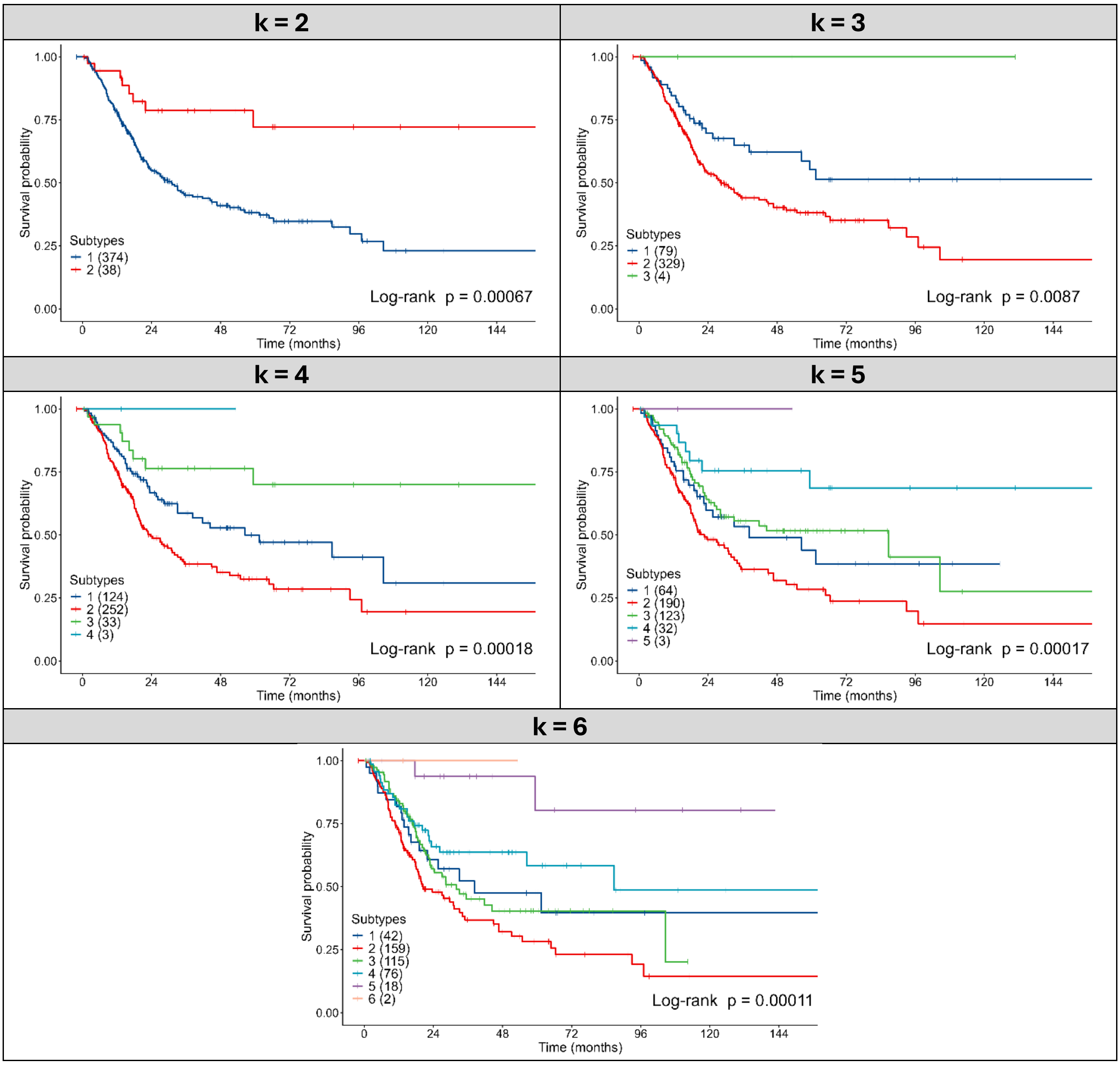

Supplementary Figure S8. Kaplan–Meier survival curves for ovarian cancer patients for different values of k with STRING network.

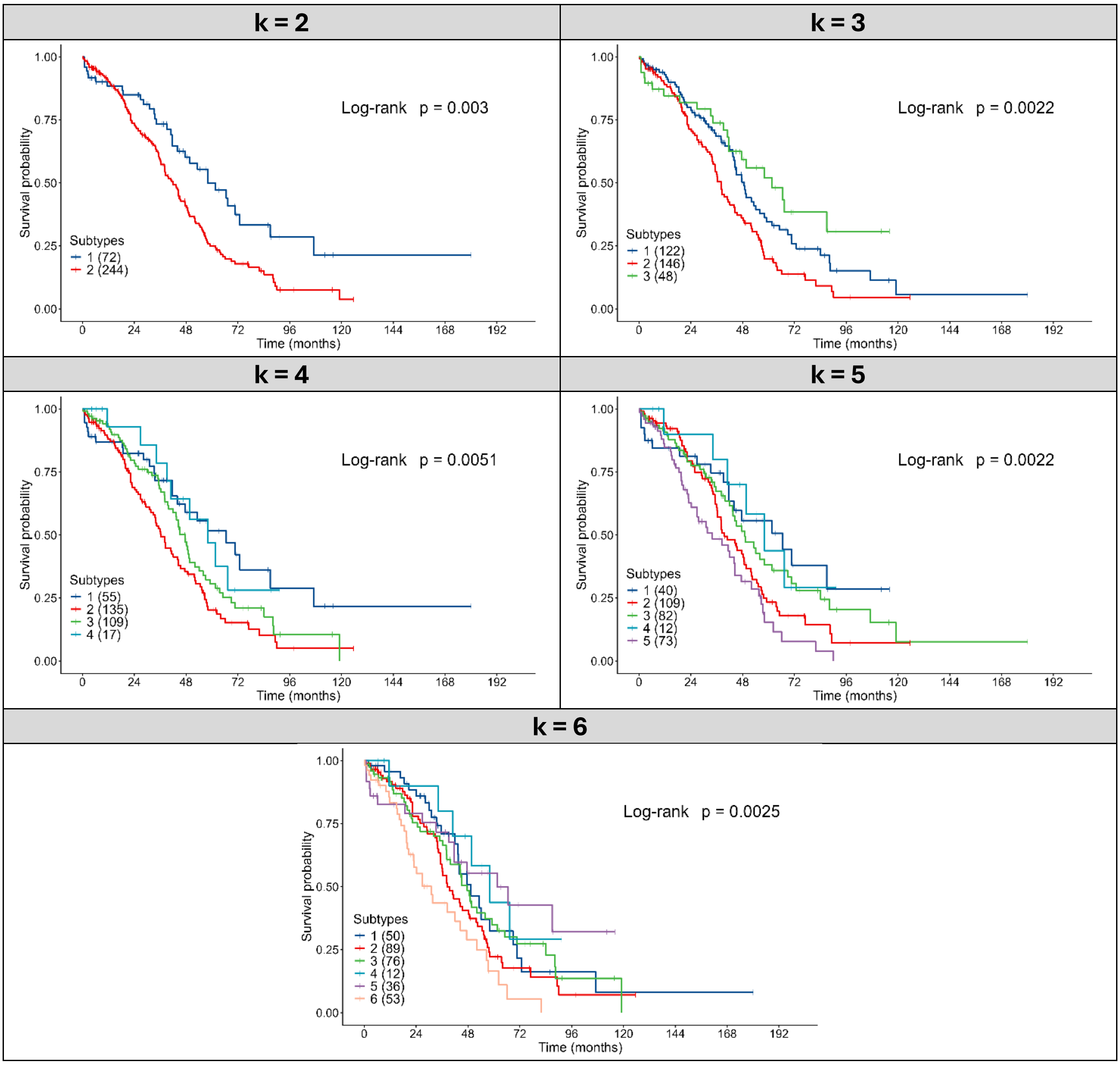

Supplementary Figure S9. Kaplan–Meier survival curves for ovarian cancer patients for different values of k with HumanNet network.

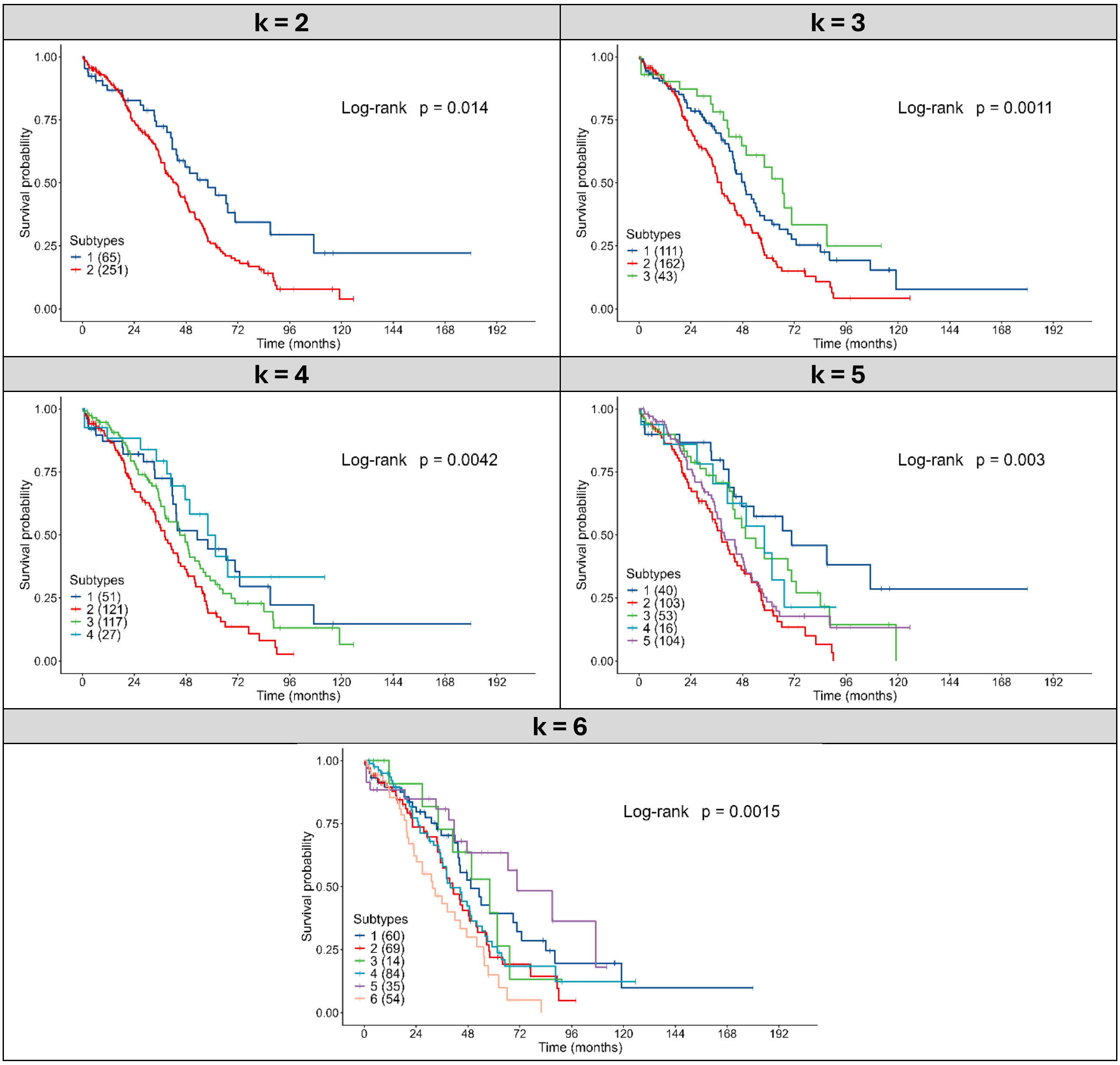

Supplementary Figure S10. Kaplan–Meier survival curves for ovarian cancer patients for different values of k with Mentha network.

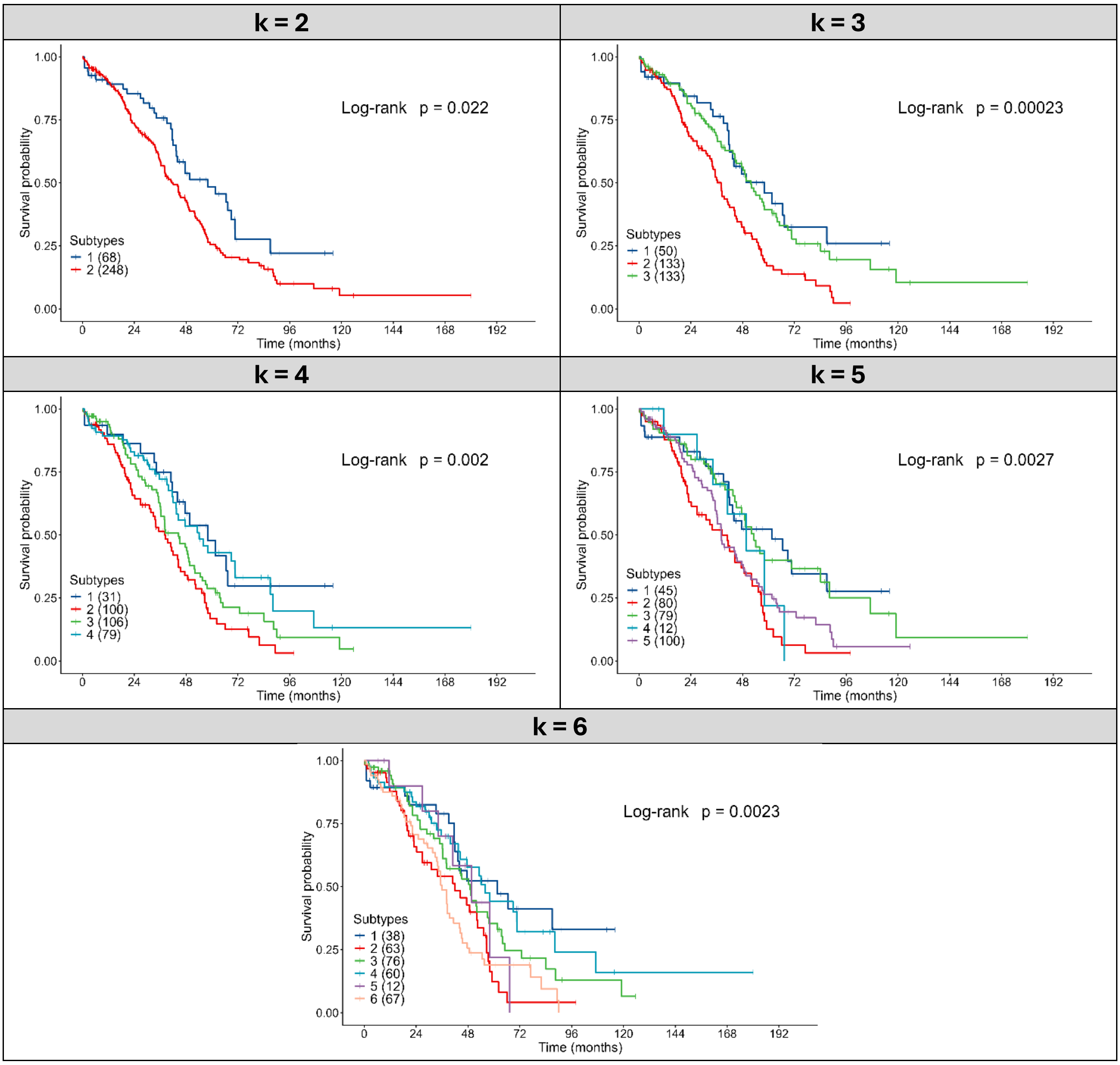

Supplementary Figure S11. Kaplan–Meier survival curves for kidney cancer patients for different values of k with STRING network.

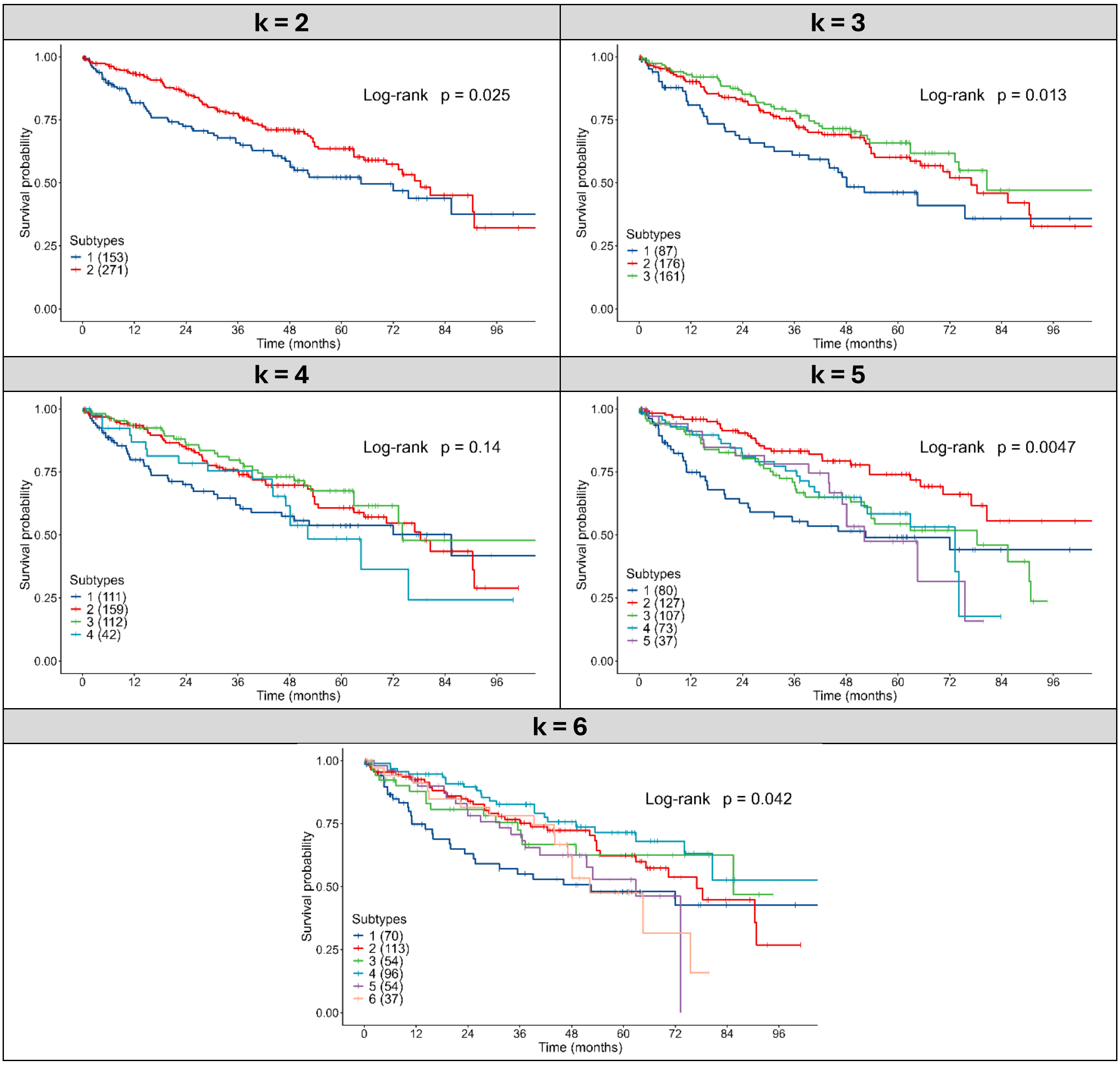

Supplementary Figure S12. Kaplan–Meier survival curves for kidney cancer patients for different values of k with HumanNet network.

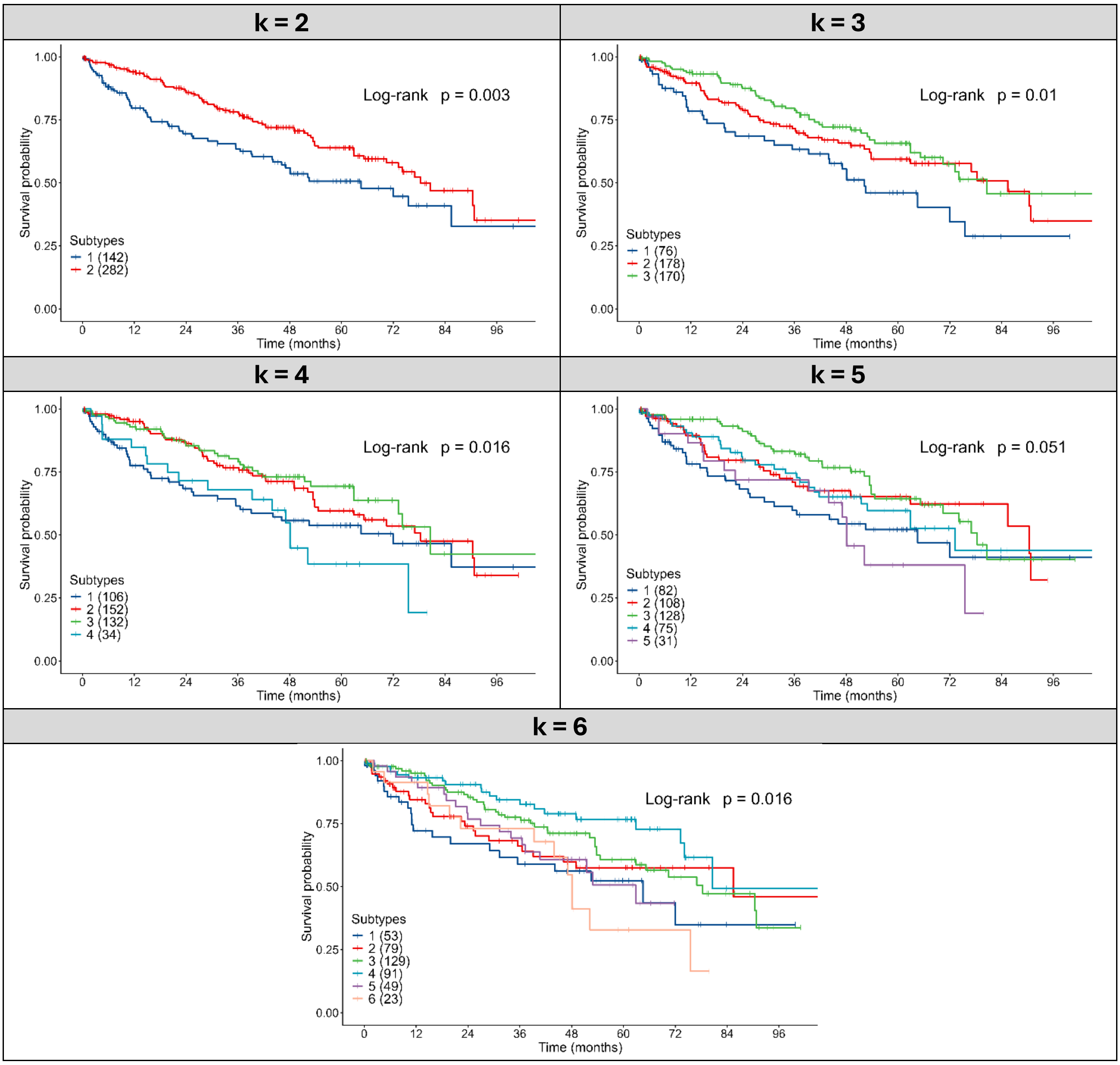

Supplementary Figure S13. Kaplan–Meier survival curves for kidney cancer patients for different values of k with Mentha network.

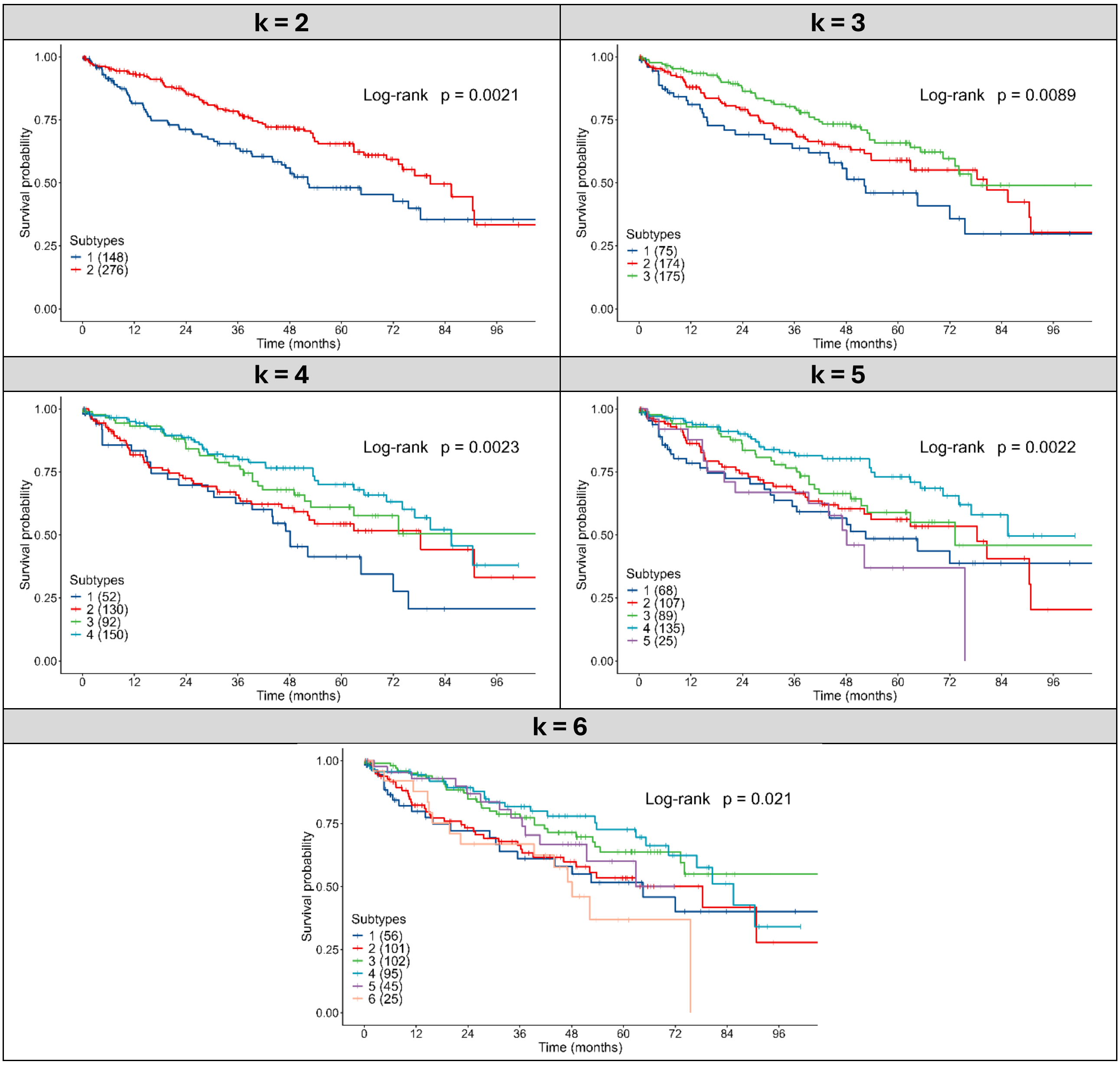

Supplementary Table S1. Univariable Cox proportional hazard models of the characteristics for OS (BLCA data with k = 4).

**Characteristic**	**N**	**Event N**	**HR**[1]	**95% CI**[1]	**p-value**	**p-value**[2] **global**
Subtype (k)	409	179				**<0.001**
1			1.99	0.93, 4.25	0.074	
2			3.60	1.75, 7.38	**<0.001**	
3 *(reference)*			—	—		
4			0	— , —	0.994	
Sex	409	179				
Female *(reference)*			—	—		
Male			0.89	0.64, 1.23	0.480	
Age at diagnosis	409	179	1.03	1.02, 1.05	**<0.001**	
Weight	366	156	1.00	0.99, 1.01	0.897	
Tumor Stage	407	178				**<0.001**
I-II *(reference)*			—	—		
III			1.61	1.05, 2.45	**0.028**	
IV			2.98	2.01, 4.41	**<0.001**	
Grade	406	179				
High Grade *(reference)*			—	—		
Low Grade			0.34	0.09, 1.39	0.135	
Histological subtype	404	175				
Non-Papillary *(reference)*			—	—		
Papillary			0.68	0.48, 0.96	**0.031**	

[1]HR = Hazard Ratio, CI = Confidence Interval. [2]global p-value for categorical variables with more than two categories. Bold values indicate a p-value < 0.05.

Supplementary Table S2. Distribution of the number of mutated genes per patient in each subtype and in the whole population for bladder cancer, with k = 4.

		**N mutated genes**	
**Cohort**	**N**	**Mean [Range]**	**Median [IQR]**
Whole Population	412	203 [1; 2592]	146 [81; 255]
Subtype 1	121	275 [170; 440]	261[214; 328]
Subtype 2	255	99 [1; 192]	95 [64.5;136]
Subtype 3	34	615 [434; 1144]	572 [497; 706]
Subtype 4	2	2002 [1413; 2592]	2002 [1708; 2297]

Range = [min; max]. IQR = Interquartile Range [Q1; Q3].

Supplementary Table S3. Univariable Cox proportional hazard models of the characteristics for OS (OVCA data with k = 3).

Characteristic	N	Event N	HR[1]	95% CI[1]	p-value	p-value[2] global
Subtype (k)	315	181				**<0.001**
1			1.42	0.84, 2.38	0.188	
2			2.17	1.33, 3.55	**0.002**	
3 *(reference)*			—	—		
Age at diagnosis	315	181	1.02	1.00, 1.03	**0.011**	
Tumor stage	314	180				0.129
II *(reference)*			—	—		
III			2.06	0.84, 5.03	0.112	
IV			2.42	0.94, 6.22	0.068	
Grade	308	176				
G2			—	—		
G3			1.44	0.86, 2.42	0.163	
Residual tumor after surgery	278	167				
> 10mm			—	—		
≤ 10mm			0.73	0.52, 1.02	0.067	
Response after platinum therapy	260	144				**<0.001**
Complete Response *(reference)*			—	—		
Partial Response			3.65	2.34, 5.70	**<0.001**	
Progressive Disease			5.24	3.24, 8.48	**<0.001**	
Stable Disease			3.12	1.50, 6.50	**0.002**	

[1]HR = Hazard Ratio, CI = Confidence Interval. [2]global p-value for categorical variables with more than two categories.
Bold values indicate a p-value < 0.05.

Supplementary Table S4. Distribution of the number of mutated genes per patient in each subtype and in the whole population for ovarian cancer, with k = 3.

		N mutated genes	
Cohort	N	Mean [Range]	Median [IQR]
Whole Population	316	44 [6; 152]	38 [28; 54]
Subtype 1	111	51 [35; 74]	49 [44; 57.5]
Subtype 2	162	26 [6; 42]	28 [20; 33]
Subtype 3	43	92 [68; 152]	84 [78.5; 105.5]

Range = [min; max]. IQR = Interquartile Range [Q1; Q3].

Supplementary Table S5. Univariable Cox proportional hazard models of the characteristics for OS (KIRC data with k = 2).

Characteristic	N	Event N	HR[1]	95% CI[1]	p-value	p-value[2] global
Subtype (k)	421	142				
1			1.68	1.20, 2.34	**0.002**	
2 *(reference)*			—	—		
Sex	421	142				
Female *(reference)*			—	—		
Male			0.83	0.59, 1.16	0.281	
Age at diagnosis	421	142	1.04	1.02, 1.05	**<0.001**	
Tumor stage	420	141				**<0.001**
I-II *(reference)*			—	—		
III			2.78	1.83, 4.23	**<0.001**	
IV			6.33	4.23, 9.48	**<0.001**	
Grade	420	141				**<0.001**
G1-G2 *(reference)*			—	—		
G3			1.52	1.01, 2.28	**0.043**	
G4			4.47	2.90, 6.89	**<0.001**	

[1]HR = Hazard Ratio, CI = Confidence Interval. [2]global p-value for categorical variables with more than two categories. Bold values indicate a p-value < 0.05.

Supplementary Table S6. Distribution of the number of mutated genes per patient in each subtype and in the whole population for kidney cancer, with k = 2.

		N mutated genes	
**Cohort**	**N**	**Mean [Range]**	**Median [IQR]**
Whole Population	424	49 [8; 121]	46 [35; 60]
Subtype 1	148	68 [37; 121]	66 [56.75; 78]
Subtype 2	276	38 [8; 67]	38 [31; 46.25]

Range = [min; max]. IQR = Interquartile Range [Q1; Q3].
